# Nutritional status of school children living in Northern part of Sri Lanka

**DOI:** 10.1186/s12887-021-02501-w

**Published:** 2021-01-19

**Authors:** M. G. Sathiadas, Annieston Antonyraja, Arunath Viswalingam, Kasthuri Thangaraja, V. P. Wickramasinghe

**Affiliations:** 1 Department of Paediatrics, University of Jaffna, PO Box: 57, Adiyapatham Road, Jaffna, Sri Lanka; 2 Department of Paediatrics, Faculty of Medicine, University of Colombo, Colombo, Sri Lanka

**Keywords:** Jaffna, Nutritional status, Obesity, Sri Lanka, Stunting, Wasting

## Abstract

**Background:**

Nutritional status is an important indicator for measuring quality of life in children. A region that is recovering from war will face many problems related to nutrition. Very few studies have addressed the nutritional problems in school children. This study was undertaken to identify the prevalence of wasting, stunting and obesity among school children from Northern Sri Lanka and associated socio-demographic factors.

**Methods:**

A community based cross-sectional study was carried out using multistage stratified proportionate cluster among healthy children attending schools in the Northern part of the country. Height and weight were measured, and Body Mass Index (BMI) calculated [weight (kg)/Height (m) ^2^]. BMI-for-age z-score (BAZ) and Height for age Z (HAZ) scores were determined and WHO growth references were used to categorise the nutritional status. Correlation between various nutritional problems with Maternal education, household income, number of family members and the residential area was assessed.

**Results:**

A total of 1012 children were recruited, and the mean age and standard deviation were 11.12±1.77 yrs. Girls in the age ranges of 9–14 were heavier and taller when compared to the boys compatible with the pubertal growth spurt. Stunting based on the height for age was seen in 10.9% of boys and 11.8% of girls. Wasting based on BMI for age WHO standard (WHO 2007) was seen in 30.6% of boys and 29.1% of the girls. The prevalence of overweight was 11% and Obesity was 6.3% of the population. Obesity was predominantly seen in boys (4.2%) and it was significantly higher when compared to the girls (2.1%) (*p* < 0.001). Obesity in older boys (> 10 years) was significantly more than the younger ones (*p* < 0.01). Maternal education and family income had a significant impact on the prevalence of wasting, stunting and obesity whereas the family size contributed to the wasting and obesity (*p*< 0.001).

**Conclusion:**

Findings suggest that stunting, wasting, overweight and obesity are prevalent among 6–16-year-old leading to concerns in public health. The nutritional status significantly varies according to the geographical location, maternal education and the household income.

**Supplementary Information:**

The online version contains supplementary material available at 10.1186/s12887-021-02501-w.

## Background

Nutritional status is an important indicator for measuring quality of life in children. School age is identified as a dynamic age for physical growth and mental development. Malnutrition manifests in many ways ultimately affecting growth and development. A country that faces serious public health challenge from malnutrition would experience adverse economic outcomes [1]. It has been shown that 11% of Gross Domestic Product (GDP) every year in Africa and Asia is spent on treating and managing problems related to malnutrition, whereas preventing malnutrition delivers USD16 in returns on investment for every USD1 spent [2].

A literature search was done in PubMed using the keywords and most have addressed under nutrition in children under-5-year of age. Very few studies have addressed nutritional issues of school-aged children to assess the magnitude of the problem [3]. In several countries the coexistence of the paradoxical situation of adult obesity with childhood malnutrition has been noted. Number of studies has shown that many households, particularly in middle-income countries, contain both underweight and obese individuals, which is described as the ‘dual burden’ of malnutrition [4].

The most important nutritional problem in the world today is obesity and it is rising rapidly especially in the developing countries. In 2018 almost 40 million under five-year old children were found to be overweight or obese. In 2016 over 340 million children and adolescents were either overweight or obese [5]. The 2015 Annual Health Bulletin of Sri Lanka reported that the prevalence of wasting was 20.3% and stunting 8.7% among primary school children [6]. However, national data lacks prevalence of overweight and obesity. The prevalence of obesity has increased rapidly in almost all countries, and these country-specific trends are now coalescing to create a true pandemic. This pandemic is penetrating the poorest nations in the world mainly among the urban middle-aged adults and younger age groups [3]. Childhood obesity is also increasing in Sri Lanka with 14–15% prevalence of overweight and obesity among 8–12-year-old school children within the Colombo Municipal area and the world bank report in 2016 estimates that 2% of the under five-year-old children to be affected [7, 8].

Obesity is caused by an imbalance between energy intake and energy expenditure. The relative contribution of physical activity, sedentary life and diet, to the development of obesity in children is unclear, partly because these variables are difficult to measure, and the metabolism of energy in the body is complex. Linear growth failure in childhood is the most prevalent form of under nutrition globally. Around 165 million children under 5 years of age are stunted. World Health Assembly has identified stunting as a major global health priority and the target is to reduce stunting by 40% between 2010 and 2030 [9]. Stunting has shown to have a high morbidity and mortality especially with infectious diseases like pneumonia and diarrhea. They also demonstrated high incidence of Tuberculosis, hepatitis and cellulitis indicating association with generalized immune suppression [10]. Furthermore, stunting has contributed to the increase prevalence of overweight especially when growth monitoring is done solely based on weight measurements.

A study done in the Western province of Sri Lanka suggested that prevalence of obesity was higher among boys (4.3%) than in girls (3.1%). The prevalence of thinness was 24.7% in boys and 23.1% in girls. Stunting was seen in 5.1% of boys and 5.2% of girls [7, 8]. As there are no other major studies including large numbers this study will help the policy makers to identify important nutritional problems in this region. This region is recovering from a 30 year old civil war and this study will help the different stakeholders to make the decision on the interventions especially the problem of obesity and it’s metabolic derangements can be dealt with early in life.

The current accepted parameters for the assessment of nutritional status in older children and adolescents are height for age z scores and Body Mass Index (BMI) z scores based on WHO references [5]. Most large-scale studies have used BMI to identify adiposity in children as well as monitoring overweight and obesity in the paediatric practice [11]. As currently used BMI cutoff does not reflect the true picture of adiposity, attention has been given to the measurement of body composition as a rise in obesity related metabolic derangements are seen in children with lower BMI values [11]. Body composition mainly measure the body fat mass and the fat free mass using dual-energy X-ray absorptiometry, stable isotope dilution and total body electrical conductivity [12].

Maternal education and socio-economic status have been proven contributing factors for childhood nutritional problems. Ability to acquire the needed knowledge to practice feeding among the main carers is important to prevent nutritional problems. Time and effort spent on childhood feeding practices also has contributed to nutritional status of the children [3].

The objective of this study was to identify the prevalence of wasting, stunting and obesity among school children from Northern Sri Lanka and associated socio-demographic factors.

## Methods

### Setting

A community based cross-sectional study was carried out using multistage stratified proportionate cluster among 6–16-year-old healthy children attending schools in the Northern part of the country. The study period was November 2015 to August 2016. The sample size was calculated using the expected proportion of children with malnutrition of 14%; level of precision of 0.03; confidence interval of 0.05; and a non-repose rate of 10% [13].

### Study population

The district of Jaffna has a mixed population, and they are clustered into various zones. The educational sector is divided into five zones based on the geographical location. Schools in all the five educational zones were considered.

Girls and boys aged 6–16 years attending secondary schools (Schools that include grade 1–10) were selected using multistage stratified proportionate cluster sampling. All five educational zones namely Jaffna, Valikamam, Vadamaradchi, Thenmaradchi and the Islands were considered. Ten schools located in the district of Jaffna was selected using a recently updated list of secondary schools in the district of Jaffna with a probability proportionate to the size of student population in each educational zone and the school. Then within each selected school, one class from each grade of one to ten (classes that have at least 10 students) was included as the cluster. The entire class was considered as a cluster and sampled.

Eligible students were informed and written consent from the parents and accent from the children was obtained. Prior permission was obtained from the relevant authorities at the educational and health sector. General information regarding socio-demography, educational background of the parents, family income, details of siblings and details of the housing were collected using a pretested questionnaire designed by the authors and validated. Children not of Sri Lankan origin or who have not been living in Sri Lanka during the last 5 years or were suffering from long-term illness were excluded.

### Assessment of nutritional status

Anthropometric measurements of students were performed at each school premises by a trained team consisting of medical graduates. The weight was measured using an electronic weighing scale to the closest 100 g wearing light clothing. The height was measured using a stadiometer to the last completed 0.1 cm without shoes, with the occiput, back of chest, buttocks and heel touching the vertical plane and head in the horizontal Frankfurt plane. Body Mass Index (BMI) was calculated [weight (kg)/Height (m) ^2^] and BMI-for-age z-score (BAZ) were used to determine the nutritional status. WHO growth references for children aged 6–16 years old was used for categorisation. Wasting was defined where BAZ less than − 2 standard deviations (SD) from the median (BAZ < − 2), overweight was defined where BAZ was more than + 1SD and less than + 2SD from the median (BAZ + 1−+ 2). Obesity was defined if BAZ was greater than + 2 SD above the median (BAZ > + 2). Based on the height-for-age Z-score (HAZ) stunting was defined as less than − 2 SD (HAZ<-2SD) [5].

Maternal education, household income, family size and residential area were used as indicators of the socio-economic status of the family. Maternal education was categorised as low when they reported as no education, primary-incomplete, primary-compete. Good education was considered as secondary-incomplete, secondary-complete, and higher degree. For household income, participants were asked to select their monthly average income in LKR (USD 1=LKR 150) from the following categories: low income if LKR< 15,000 and adequate incomes if > 15,000. Family size more 5 was considered as a large family size. Residential area was considered as rural or urban.

### Data analysis

Data was entered in Microsoft excel 2007 and analysed by using Statistical Package for Social Sciences (SPSS) software version 22.0. Z scores were calculated using software. One-way ANOVA was used to compare the means of height, weight, and BMI. Chi square was used to assess the difference of obesity between males and females as well as compared the older males (age> 10 yrs) and younger males (age< 10 yrs). Chi square was used to assess the different nutritional problems in different education zones. The potential association between demographic characteristics and the nutritional status among children, was investigated using the odds ratio and 95% Confidence Interval and statistically significant was considered when the *p* value was < 0.05.

Ethical Review Committee of the Faculty of Medicine, University of Jaffna approved the study (J/ERC/14/50/NDR/0077) and Permission for the study was also obtained from the Department of Education, Northern Province of Sri Lanka.

## Results

A total of 1012 children (496 Boys) from all five educational zones were recruited. The children were categorized based on the educational zone and age. Table 1 shows the socio-demographic distribution of the population.

**Table 1:** Socio-demographic pattern of the study population

Socio demographic details		Number of Students (%)
Gender	Males	496 (49)
	Females	516 (51)
Mean Age (Yrs) ±SD	Males	11.11±1.76
	Females	11.13±1.88
Educational zones (%)	Jaffna	320 (31.6)
	Vadamaradchi	234 (23.1)
	Thenmaradchi	204 (20.2)
	Valikamam	103 (10.1)
	Islands	151 (15.0)
Maternal Age (years)	< 20	54 (5.3)
	20–29	297 (29.3)
	30–39	355 (35)
	40–49	234 (23.1)
	> 50	72 (7.1)
Household income ^a^	Low	320 (31.6)
	Middle	446 (44)
	High	246 (24.3)
Maternal Educational Level	Nil	10 (1)
	Incomplete primary	24 (2.4)
	Complete primary	176 (17.4)
	Incomplete secondary	402 (39.7)
	Complete Secondary	340 (33.6)
	Higher degree	60 (5.9)

^a^Household equivalent income (HEI): low (LKR< 15,000), middle (LKR 15,000–< 50,000), or high (LKR ≥50,000)

Mean heights of girls 9 through 13 years were higher than that of same age boys, which could be due to the pubertal growth spurt, occurring in girls earlier than boys. Similarly, the 9–13-year-old girls were slightly heavier than the boys and it reverses after the age of 14 years. Table 2 gives the mean height, weight, and BMI along with the Z scores for both boys and girls. One-way ANOVA showed the differences in the means between boys and girls was not significant (Table 2). Significant difference was not seen when the anthropometric parameters were segregated according to educational zones. The mean Z scores for the height was below the median except for 8 year old girls and boys. The mean Z scores for the weight and BMI of all ages in both gender groups were below the median. (Table 2).

**Table 2:** Mean height, weight and BMI the children according to the age and sex

Age (yrs)	N		^a^Height				^b^Weight				^c^BMI			
			Mean±SD (cm)		Mean Z score ±SD		Mean±SD (kg)		Mean Z score ±SD		Mean±SD (kgm^− 2^)		Mean Z score ±SD	
	Male	Female	Male	Female	Male	Female	Male	Female	Male	Female	Male	Female	Male	Female
6	**20**	**21**	110.9±3.7	109.6±2.8	−1.0 ±0.7	− 1.1±0.5	17.2±2.9	15.9±1.2	−0.2±1.2	−0.4±1.1	13.9±1.5	13.2±0.9	−1.2±1.1	−1.5±0.8
7	**17**	**17**	116.7±5.4	114.9±5.6	−0.9±1.0	−1.08±1.0	18.9±3.0	17.9±3.1	−0.7±1.5	−0.4±1.1	13.9±2.8	13.5±1.9	−1.5±1.8	−1.5±1.5
8	**30**	**31**	126.7±6.5	125.2±5.9	0.9±1.1	−0.21±1.0	22.6±3.9	21.5±3.8	0.7±0.9	−0.3±1.0	14.0±1.4	13.6±1.6	−1.4±1.1	−1.5±1.3
9	**40**	**42**	128.1±6.3	125.9±19.3	−0.7±1.0	−0.6±1.01	23.2±4.8	23.9±6.3	0.4±0.7	0.2±0.9	14.0±2.02	14.6±2.5	−1.7±1.4	−1.1±1.5
10	**47**	**49**	134.6±6.4	135.4±8.7	−0.5±1.0	−0.5±1.3	28.5±7.2	28.8±10.	−0.9±1.1	0.1±1.1	15.5±2.9	15.4±3.8	−0.9±1.7	−1.1±1.5
11	**72**	**76**	141.3±6.8	143.1±8.7	−0.2±1.0	−0.3±1.3	32.6±8.6	33.5±9.8	−0.5±1.3	−0.5±1.4	16.1±3.2	16.1±3.0	−0.9±1.7	−0.9±1.6
12	**75**	**78**	143.6±7.1	144.8±6.8	−0.7±1.0	−0.9±1.0	35.0±10.2	35.2±9.5	−0.5±1.6	−0.7±1.4	16.7±3.8	16.5±3.5	−0.9±1.8	−1.1±1.6
13	**74**	**76**	147.4±7.5	151.0±1.3	−1.1±1.0	−0.7±1.0	35.3±11.3	41.1±10.9	−1.2±1.4	−0.5±1.4	16.0±3.6	17.8±3.8	−1.7±1.6	−0.8±1.6
14	**50**	**52**	156.4±8.8	155.2±6.3	−0.8±1.1	−0.6±0.9	45.2±15.3	45.5±10.4	−0.7±1.7	−0.6±1.4	18.1±4.7	18.8±3.7	−1.0±2.1	−0.6±1.4
15	**43**	**45**	163.2±9.7	157.1±7.3	−0.7±1.2	−0.6±1.0	49.4±13.4	45.9±10.9	−0.7±1.4	−1.1±1.6	18.4±4.2	18.5±3.8	−1.1±1.8	−1.0±1.6
16	**28**	**29**	169.3±8.2	157.9±7.1	−0.4±1.1	−0.7±1.0	57.2±15.4	50.4±10.2	−0.2±1.5	−0.4±1.2	19.7±4.4	20.1±3.4	−0.7±1.8	−0.4±1.1
Total	**496**	**516**	143.0±16.2	142.3±15.8	−0.7±1.1	−0.6±1.1	34.8±14.6	34.9±13.2	−0.5±1.4	−0.5±1.3	16.3±3.8	16.6±3.7	−1.2±1.7	−0.9±1.5

^a^One way ANOVA F (1,22)=0.05 *p* = 0.8, ^b^F (1,22)=0.008 *p* = 0.9, ^c^F (1,22)=0.041 *p* = 0.84

Prevalence of stunting was in 10.9% of boys and 11.8% of girls. Wasting was seen in 30.6% of boys and 29.1% of the girls (Table 3).

**Table 3:** Prevalence nutritional status according to age and sex

Age	No		Height for age				BMI for Age															
			Stunting				Wasting				Normal				Overweight				Obese ^a, b^			
	Male	Female	Male		Female		Male		Female		Male		Female		Male		Female		Male		Female	
			Ν	***%***	Ν	%	Ν	%	Ν	%	Ν	%	Ν	%	Ν	%	Ν	%	Ν	%	Ν	%
6	**20**	**21**	**2**	*10.0*	**2**	*9.5*	**07**	*35.0*	**13**	*62.0*	**12**	*60.0*	**7**	*33.3*	**1**	*5.0*	**1**	*4.7*	**0**	*0*	**0**	*0*
7	**17**	**17**	**2**	*11.7*	**3**	*17.6*	**07**	*41.2*	**8**	*47.0*	**09**	*53.0*	**6**	*35.3*	**0**	*0*	**2**	*11.8*	**1**	*5.9*	**1**	*5.9*
8	**30**	**31**	**1**	*3.3*	**1**	*3.2*	**09**	*30.0*	**10**	*32.3*	**17**	*56.7*	**19**	*61.3*	**2**	*6.7*	**1**	*3.2*	**2**	*6.7*	**1**	*3.2*
9	**40**	**42**	**5**	*12.5*	**7**	*16.7*	**10**	*25.0*	**13**	*31.0*	**22**	*55.0*	**25**	*59.5*	**4**	*10.0*	**3**	*7.1*	**4**	*10.0*	**1**	*2.4*
10	**47**	**49**	**6**	*12.8*	**6**	*12.2*	**14**	*29.8*	**12**	*24.5*	**23**	*49.0*	**31**	*63.3*	**5**	*10.6*	**5**	*10.2*	**5**	*10.6*	**1**	*2.0*
11	**72**	**76**	**1**	*1.4*	**4**	*5.3*	**26**	*36.1*	**20**	*26.3*	**34**	*47.2*	**46**	*60.5*	**8**	*11.1*	**8**	*10.5*	**4**	*5.5*	**2**	*2.6*
12	**75**	**78**	**9**	*12.0*	**6**	*2.6.0*	**27**	*36.0*	**22**	*28.2*	**37**	*49.3*	**47**	*60.3*	**7**	*9.3*	**7**	*9.0*	**4**	*5.3*	**2**	*2.6*
13	**74**	**76**	**10**	*13.5*	**10**	*13.1*	**20**	*27.0*	**21**	*27.6*	**35**	*47.3*	**42**	*55.3*	**8**	*10.8*	**10**	*13.2*	**11**	*14.9*	**3**	*3.9*
14	**50**	**52**	**8**	*16.0*	**5**	*9.6*	**15**	*30.0*	**13**	*25.0*	**23**	*46.0*	**28**	*53.8*	**7**	*14.0*	**9**	*17.3*	**5**	*10.0*	**2**	*3.8*
15	**43**	**45**	**5**	*11.6*	**11**	*24.4*	**11**	*25.6*	**11**	*24.4*	**22**	*51.1*	**24**	*53.3*	**6**	*13.9*	**7**	*15.5*	**4**	*9.3*	**3**	*6.6*
16	**28**	**29**	**5**	*17.9*	**6**	*20.7*	**06**	*21.4*	**7**	*24.1*	**11**	*39.3*	**14**	*48.3*	**8**	*28.6*	**3**	*10.3*	**3**	*10.7*	**5**	*17.2*
Τοτ	**496**	**516**	**54**	*10.9*	**61**	*11.8*	**152**	*30.6*	**150**	*29.1*	**245**	*49.4*	**289**	*56.0*	**56**	*11.3*	**56**	*10.7*	**43**	*8.7*	**21**	*4.1*

^a^Boys were significantly obese than girls X^2^(11012)=9.03 *p* < 0.002

^b^Older Boys (> 10 years) were significantly obese than younger boys (< 10 years) X^2^(1496)=4.79 *p*< 0.02

The prevalence of overweight was 11% and obesity was 6.3% of the population. Overweight was predominantly seen in 9–14-year-old girls and those were the most affected ages. Obesity was predominantly seen in boys (8.7%) and it was significantly higher when compared to the girls (4.1%) (*p* < 0.001). Older boys (> 10 years) were significantly affected than the younger ones (*p* < 0.02) (Table 3).

The prevalence of the types of malnutrition in the different educational zones suggested that Thenmaradchi zone had a significantly higher prevalence of stunting (*p*< 0.001) and obesity and overweight were more prevalent in the Jaffna zone (*p*< 0.001) (Table 4). The islands and Thenmaradchi zones had very minimal prevalence of obesity and overweight.

**Table 4:** Prevalence of malnutrition according to educational zones

Educational Zones	N	Stunting N (%)^a^	Wasting N (%)^b^	Overweight N (%)^c^	Obese N (%) ^c^
Jaffna	320	23 (20)	81 (26.8)	38 (34)	22 (34.4)
Vadamaradchi	234	19 (16.5)	45 (14.9)	28 (25)	16 (25)
Thenmaradchi	204	34 (29.5)	88 (29.1)	10 (8.9)	08 (12.5)
Valikamam	103	23 (20)	54 (17.9)	22 (2.0)	12 (18.8)
Islands	151	16 (14)	34 (11.3)	14 (12.5)	06 (9.4)
Total	1012	115 (11.3)	302 (29.8)	112 (11.1)	64 (6.3)

Prevalence of malnutrition within the zones X^2^(12, 1012) =31.95 *p* < 0.001

^a^Stunting X^2^(41012) =26.07 *p* < 0.001, ^b^Wasting X^2^(41012)=61.9 *p* < 0.0001, Overweight and obesity X^2^(41012)=30.45 *p* < 0.0001

Maternal Education and Family income had a significant impact on the prevalence of stunting, wasting, overweight and obesity (*p*< 0.001). Family size had a significant impact on the wasting, overweight and obesity. The residential area had a significant impact on the stunting but not in wasting, overweight and obesity. Table 5 shows the comparative date on the nutritional problems and the socio-demographic factors.

**Table 5:** Association of socio-demographic factors to nutritional problems

Socio-economic factors	Stunting			Wasting			Overweight and obesity		
	Yes (%)	No (%)	OR (95%CI) *P* Value	Yes (%)	No (%)	OR (95%CI) *P* Value	Yes (%)	No (%)	OR (95%CI) *P* Value
Education level of the mother			7.79			3.23			2.87
Low	62 (54)	117 (13)	(5.15–11.8)	79 (26)	70 (10)	(2.26–4.62)	51 (29)	104 (12)	(1.95–4.33)
Good	53 (46)	780 (87)	< 0.001	223 (74)	640 (90)	< 0.001	125 (71)	732 (88)	< 0.0001
Family Income			61.4			22.71			16.07
Low	103 (89)	110 (12)	(32.69–115-3)	245 (81)	113 (16)	(15.97–32.27)	124 (70)	108 (13)	(10.97–23.54)
Adequate	12 (11)	787 (88)	< 0.0001	57 (19)	597 (84)	< 0.001	52 (30)	728 (87)	< 0.0001
Family Size			0.72			0.38			0.18
< 5	105 (91)	839 (94)	(0.36–1.4)	194 (64)	584 (82)	(0.28–0.52)	102 (58)	738 (88)	(0.12–0.26)
> 5	10 (9)	58 (6)	= 0.3	108 (36)	126 (18)	< 0.0001	74 (42)	98 (12)	< 0.0001
Residential Area			2.4			0.87			0.84
Rural	75 (65)	429 (48)	(1.36–3.06)	109 (36)	290 (41)	(0.62–1.08)	59 (34)	313 (37)	(0.59–1.18)
Urban	40 (35)	468 (52)	= 0.0005	193 (64)	420 (59)	= 0.15	117 (66)	523 (63)	= 0.3

^a^Low was no education, incomplete and complete primary. Adequate was incomplete secondary, secondary and higher degree

^b^Household equivalent income (HEI): low less than LKR 15,000, adequate more than LKR 15,000

## Discussion

Malnutrition is a universal problem, and the impact varies between regions and countries. Out of the eight key nutrition indicators to track the progress of malnutrition, three are in children namely childhood stunting, wasting and obesity [14]. The WHO sustainable development goal targets for 2025 are to reduce stunting in childhood by 40%, no increase in obesity and reduce and maintain childhood wasting to less than 5% [9]. Nutritional problems are multiple, and it is clearly seen from the results of our study as well.

Stunting in early childhood has shown to increases the risk of dying from childhood illnesses, impairs cognitive development, lower the educational performance and reducing job opportunities later in life. Stunting can be accompanied by later excessive weight gain thus increasing the risk of developing obesity and NCD as adults. According to the National surveys and UNICEF/WHO/World Bank Group Joint Child Malnutrition report, it is estimated that 35% of the children would be stunted in the South-East Asian region and 17.3% in Sri Lanka [14]. Our study done in a single district in the North shows stunting in 10.9% of boys and 11.8% of girls. A similar study done in Colombo district showed stunting in 5.1% of boys and 5.2% of girls [7]. Low prevalence of stunting in two districts may be influenced by the demography where both these districts are urban, and the economic status and educational level of the population is higher [15].

Wasting is also a major issue in most countries in the region. Although most countries in the region have shown an economic growth, there are other factors such as traditional food habits, poor infant feeding practices, inadequate clean water and sanitation, and farming a limited variety of crops are some of the contributory factors. Globally the incidence of wasting is 7.3% and the South East region has 15% [16]. Our study showed wasting in 30.6% of the boys and 29.1% in girls. This may be due to food insecurity and lack of knowledge on healthy eating habits. Poor living standards in certain areas and risk of infections can also contribute to wasting [17].

Obesity and overweight are the identified nutritional problem that is increasing in alarming rates in this part of the world. The World Health Organization (WHO) has stated that in 2016 the number of overweight children under the age of five, is estimated to be over 41 million and the commonly affected continents are Asia and Africa [9]. The overall prevalence of overweight and obesity among school children in European countries was estimated at 20.5% [18]. The prevalence of obesity in USA was 18.5% and affected about 13.7 million children and adolescents according to the Centre for Disease Control (CDC) report from 2017 [18].

The proportion of overweight and obesity was 24.5% in Eastern Asia countries and 11.9% in the Western Asia regions [19].

A study done in the Colombo district shows a prevalence of obesity in boys was 4.3 and 3.1% in girls while our study indicated 8.7% in boys and 4.1% in girls [6]. The difference even years apart may be explained by the economic status of the populations. The prevalence of overweight and obesity combined was 17.3% and it almost matches the other Asian countries [20]. Rate of overweight and obesity and the high prevalence of under-nutrition demonstrates the existence of double burden of malnutrition in this part of the country.

A meta-analysis and systematic review done on Asian countries shows a significant higher percentage of boys (7.0%) are obese than girls (4.8%) [21]. Our data also demonstrated a significant difference between girls and boys when considering obesity. The Global Burden of Disease Study estimated that the prevalence of obesity/overweight in children and adolescents in developing countries was 12.9% for boys and 13.4% for girls in 2013 [22].

The Mean Z scores for height and weight are closer to zero hence the distribution is more homogenous. When classifying the problem of malnutrition, the WHO has defined it as low, medium and high severity based on the prevalence [23]. Prevalence of stunting < 20% indicates it as low and our study population has a low prevalence (11.3%) of stunting. Similarly, for wasting if the prevalence is < 5% it is low, 5–9% is medium, 10–14% is high and > 15 is very high. In our study population the prevalence was 29.8% indicating a very high prevalence.

There are several socio-demographic factors contributing to malnutrition in a population. Maternal literacy is an important factor for nutritional status of children in developing countries [24, 25]. We were able to demonstrate maternal educational level and family income to have a significant association with stunting, wasting, overweight and obesity. Low level of education was associated with poor nutritional status. These results were consistent with studies done in other parts of the globe [25]. Employment status of the mothers also contribute to the nutritional status of the children, but we were unable to demonstrate this fact in our population. In our study we were able to demonstrate that family size contributed to wasting, overweight and obesity but not to stunting. Birth order and family size has been shown to impact growth of children in other studies [26].

Many low- and middle-income countries have the double burden of malnutrition i.e., coexistence of problems of under nutrition and overweight particularly in the urban setting [16]. Our study also clearly demonstrates the double burden. The children in this region are vulnerable to inadequate prenatal, infant and childhood nutrition. They are also exposed to high fat, high salt, high sugar, and energy dense food, which is easily available for those who are economically stable. This problem coexists with the lower level of physical activity leading to an increase in obesity while under nutrition remains unsolved [27].

The strengths of this study are that it has been conducted in a region which has not been explored before and consists of a large cohort. The limitations are that it is confined to the Northern part and the results cannot be generalized to the rest of the country. Hence, we recommend that wider scale similar studies are needed to generalize the results to the whole country.

## Conclusion

Findings suggest that stunting, wasting, overweight and obesity are prevalent among 6–16-year-old. Low prevalence of stunting and high prevalence of wasting and presence of overweight/obesity in this population suggests public health concerns and the existence of the double burden of malnutrition. The nutritional problems significantly vary according to the geographical location, maternal education, and the household income**.** Community based interventions are recommended to prevent an increasing trend of all forms of malnutrition.

## Supplementary Information

**Additional file 1.**

## Data Availability

The data in this study is available from the corresponding author on request.

## Abbreviations

GDP: Gross Domestic Product
BMI: Body Mass Index
HAZ: Height for Age Z score
BAZ: BMI-for-age z-score
WHO: World Health Organisation
CDC: Centre for Disease Control
