# Supplementary material for: Nutritional status of school children living in Northern part of Sri Lanka
Source: BMC Pediatr. 2021 Jan 19;21:43. doi: 10.1186/s12887-021-02501-w (PMC7814636; doi:10.1186/s12887-021-02501-w)
Supplement: Supplementary file 1 — Additional file 1. [file 12887_2021_2501_MOESM1_ESM.pdf]

Serial Number:-------------------------

Nutritional status and associated demographic details in Sri Lankan children

(Filled by investigator)

1 Date of study --

2 Name --

3 Age D.O.B ---------------------------------------

4 Sex Male ☐ Female ☐

5 Address ---

----

6 Telephone Residence Mobile --------------------------------------

7 Ethnicity ---

8 Birth weight: ☐ Exclusive breast feeding: ☐ months

9 Family history of:

Obesity	Father	Mother	sibling	Grand parents	MF	MM	PF	PM
NIDDM	Father	Mother	sibling	Grand parents	MF	MM	PF	PM
HT	Father	Mother	sibling	Grand parents	MF	MM	PF	PM
IHD	Father	Mother	sibling	Grand parents	MF	MM	PF	PM
CVA	Father	Mother	sibling	Grand parents	MF	MM	PF	PM

Serial Number:-------------------------

10. **Socio Demographic and Economic factors**.

10.1. Marital status of parents

1.1. Married ☐
1.2. Unmarried ☐
1.3. Divorced ☐
1.4. Widow ☐
1.5. Separated ☐
1.6. Do not like to declare ☐

10.2. Type of family

1.7. Nuclear ☐
1.8. Extended ☐

10.3. Ethnicity

Tamil ☐
Sinhalese ☐
Muslim ☐
Burgher ☐
Other (Specify)-: ☐

10.4. Maternal education

Nil	
Incomplete primary	
Complete primary	
Incomplete secondary	
Complete Secondary	
Higher degree	

10.5 Occupation

Mothers' occupation-: --
Fathers 'occupation-: ---

Serial Number:-------------------------

10.6. Monthly family income-:

LKR<15,000 ☐
LKR 15,000–<50,000 ☐
LKR ≥50,000 ☐

10.7. Number of Siblings

Nil ☐
<5 ☐
>5 ☐

EXAMINATION

Height	---------------cm
Waist circumference	--------------- cm
Weight	----------------kg
Impedance	----------------Ω

10. Acanthosis nigricans Yes ☐ No ☐

11. Pubertal staging

Breast development	1 2 3 4 5
Pubic hair	1 2 3 4 5
Axillary hair	1 2 3

Testicular volume Left: -----ml Right: ------ml

Age of Menarche ------------------
